# Long-term end-stage renal disease risks after living kidney donation: a systematic review and meta-analysis

**DOI:** 10.1186/s12882-023-03208-z

**Published:** 2023-05-30

**Authors:** Jun Young Park, Won Jae Yang, Seung Whan Doo, Jae Joon Park, Yong Nam Gwon, Ki Min Kim, Jae Heon Kim, Do Kyung Kim

**Affiliations:** Department of Urology, Soonchunhyang University Seoul Hospital, 59 Daesagwan-Ro, Yongsan-Gu, Seoul, 04401 Korea

**Keywords:** ESRD, Living kidney donors, Systematic review, Meta-analysis

## Abstract

**Background:**

Recent studies have shown that donor nephrectomy can induce renal function impairment. However, few meta-analysis studies about this have proceeded. Therefore, the objective of this systematic review and meta-analysis including all data of recent research studies was to determine whether living donor nephrectomy (LDN) could induce renal function impairment.

**Methods:**

By November 2020, comprehensive literature searches were performed on PubMed, Embase, and Cochrane databases. Inclusion criteria were: (1) observational studies with data about overall end-stage renal disease (ESRD) or chronic kidney disease (CKD) of living kidney donors, (2) control group consisted of people without donor nephrectomy, and (3) outcomes of studies included long-term end-stage renal disease risks after living kidney donation. Risk of Bias in Non-randomized Studies of interventions (ROBINS-I) assessment tool was used to evaluate our methodological quality.

**Results:**

The qualitative review included 11 studies and the meta-analysis included 5 studies. In the meta-analysis, the integrated overall ESRD risk was 5.57 (95% CI: 2.03—15.30). Regarding the overall risk of bias using ROBINS-I assessment tool, 0 studies was rated as "Low", 7 studies were rated as "moderate", 2 studies were rated as "Serious", and two studies were rated as "Critical".

**Conclusions:**

Our study showed that LDN increased ESRD risk in LDN patients. However, in our meta-analysis, variables in included studies were not uniform and the number of included studies was small. To have a definite conclusion, meta-analyses of well-planned and detailed studies need to be conducted in the future.

**Supplementary Information:**

The online version contains supplementary material available at 10.1186/s12882-023-03208-z.

## Introduction

End-stage renal disease (ESRD) is observed in overall world, posing huge financial burden for health-care systems [1]. Kidney transplantation (KT) is known to be an ideal renal replacement therapy for ESRD [2]. Compared with patients undergoing dialysis including hemodialysis (HD) and peritoneal dialysis (PD), successful KT can improve patients’ quality of life and survival rates as well as their daily activity limitations [3, 4]. KT is typically classified as deceased-donor or living-donor transplantation. Living-donor transplantations yield much better outcomes than deceased-donor transplantations [5].

According to World Health Organization (WHO), about 39% of all KTs were living-donor KTs, with about 27.000 kidney transplantations performed annually [6]. Although death during surgery or major complication due to kidney transplantation are very rare [7], living with one kidney affects person throughout a lifetime. For patients who have undergone living donor nephrectomy (LDN), long and short term outcomes of mortality, life expectancy, quality of life, risks of ESRD, and hypertension have been assessed and validated by several studies [8]. Long-term risk of chronic kidney disease (CKD) and ESRD following LDN has been recognized [9, 10]. Impaired renal function after LDN frequently presents with augmented urine protein levels and elevated blood pressure, beyond what is anticipated from a natural aging process [9, 11].

Previously, some studies showed that the risk of ESRD in donors was not significantly high. Ibrahim et al. reported that the risk of ESRD in donors was not significantly high [12]. Rather, in large-scale case–control studies, it was not low or different from the general population. [12]. However, recent studies comparing donors to healthy non-donors on ESRD risk associated with kidney donation showed that kidney donation is related to a small but statistically significant increase in ESRD risk [13, 14].

The purpose of our study was to investigate the actual effect of LDN on long-term overall ESRD risk. In order to surmount the above issue, a retrospective cohort study was performed which included kidney donors and multiple control groups, i.e., unscreened individuals from the general population, members of the general population who had no established pathology, and healthy controls that were matched to the donor group. Risks of mortality, ESRD, and CKD were assessed. Additionally, previous systematic review had limited sample size and inappropriate comparison groups without focusing on the incidence of ESRD in living kidney donors [9]. Recently, several observational studies have been reported. They were not included in pervious systematic reviews. Therefore, we proceeded a systematic review and meta-analysis including all data of recent research studies. Risk of Bias in Non-randomized Studies of interventions (ROBINS-I) assessment tool was used to evaluate our methodological quality.

## Materials and methods

This review was conducted in accordance with the PRISMA checklist.

### Search strategy

We conducted comprehensive literature searches in PubMed, Embase, and Cochrane databases through November 2020. We set as PICO; Patient/population: living kidney donor, Intervention: living donor nephrectomy, Comparison: general population or healthy population, Outcomes: CKD including ESRD. Keywords included ‘kidney transplantation’ and ‘kidney donor’ and ‘living donor’ and ‘ESRD’ or ‘end stage of renal disease’ or ‘chronic kidney disease’. Two authors (JYP and DKK) reviewed the title and abstract in accordance with inclusion criteria independently. If authors’ opinions were different, two authors had a discussion.

### Study selection

Inclusion criteria were: (1) observational studies with data about overall ESRD or CKD of living kidney donors, (2) control group consisted of people without donor nephrectomy, (3) outcomes of studies included long-term ESRD risks after LDN. Studies without a control group were excluded. Studies were limited to English literature and conference abstracts were excluded. When duplicate studies targeting the same cohort were confirmed, the latest and appropriate results were selected through strict discussions between researchers. Two authors (JYP and DKK) independently reviewed the titles and abstracts of all articles using inclusion criteria and investigated full-text articles to ensure that they met inclusion criteria and extracted data using a data extraction forms. All investigators judged the last inclusions through discussion and evaluation. Data from the included study were cross-checked to ensure that there was no duplicate data and to improve the integrity of the meta-analysis.

### Statistical analysis

Almost all studies had a long duration and a large population size. These kinds of results are most appropriately analyzed using the risk of long-term ESRD after LDN compared to control group. Also, we divided subgroups whose outcomes were eGFR less than 60 ml/min/m^2^ versus ESRD. Two studies set outcome as eGFR less than 60 ml/min/m^2^. Three studies set outcome as ESRD which means condition that requires acute dialysis in follow-up up period. Using the random effects model published by DerSimonian and Lairdwas, we determined the overall risk ratio (HR) with a 95% confidence interval (CI) for the results. We evaluated the statistical heterogeneity using the Cochran’s Q test and the I^2^ statistics.

### Analysis of methodological quality

The ROBINS-I tool was used to assess the risk of bias in included studies. The bias caused by confounding domains was evaluated depending on whether control groups were matched or HR was adjusted. We evaluated the bias caused by selection of participant domain based on whether the control group was composed of a healthy group, a general group, or no control group. The bias due to classification of the intervention domain was considered low because all included studies included donor nephrectomy. We determined the bias due to deviations from intended interventional domains by other factors affecting donor nephrectomy such as hospital size, surgical volume, procedure type, and single/multi-center study. The bias due to missing data was evaluated in accordance with analysis of the claimed data or description of the tracking method. We evaluated the bias in measurement of outcomes domain by the presence or absence of HR, median follow-up period, and ESRD ratio. Bias in selection of the reported result was assessed according to HR, ESRD rates, and causes of ESRD.

### Analysis of heterogeneity

We evaluated the statistical heterogeneity using the Cochran’s Q test and the I^2^ statistics. Cochran’s Q statistic *P* –value < 0.1 or an I^2^ statistic index > 50% indicated significant heterogeneity between studies. Insignificant X^2^ test result (*P* ≥ 0.1) and I^2^ statistic ≤ 50% indicated a lack of evidence to support heterogeneity, but lack of statistical power to detect heterogeneity did not necessarily mean homogeneity. Thus, random effects model was used.

### Analysis of potential publication bias

Funnel plot was used to determine publication bias and without publication bias, the combined effect sizes of studies should be symmetrically distributed.

## Results

### Study selection

**Fig. 1:**
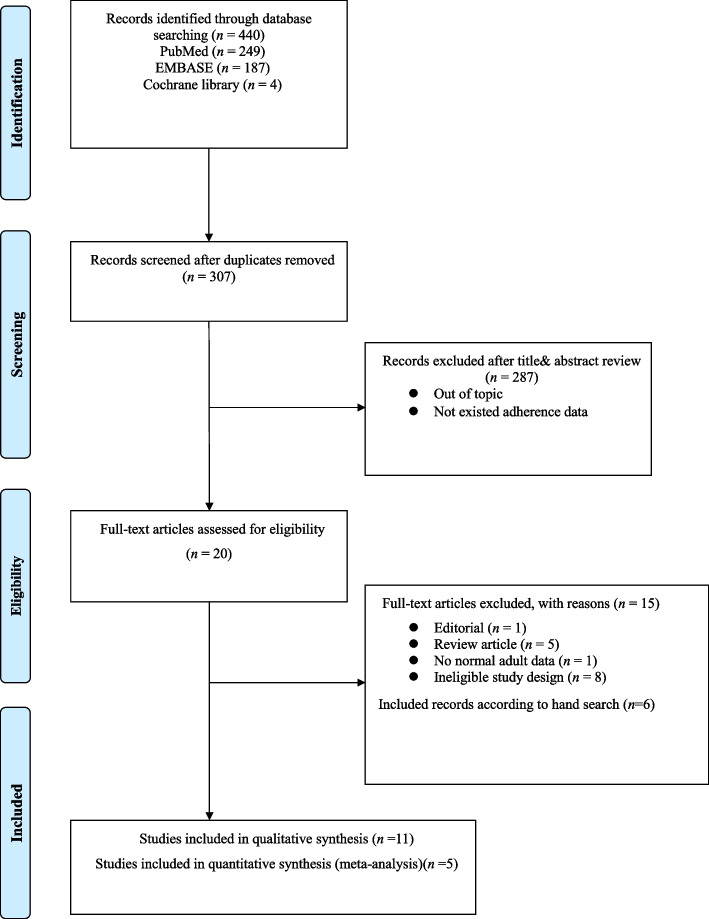
Flowchart of Preferred Reporting Items for Systematic Reviews and Meta-analysis (PRISMA)

We searched 440 articles from various electronic databases (PubMed, *n* = 249; Cochrane, *n* = 4; Embase, *n* = 187) by November 2020. 133 studies were excluded due to overlapping data or data appearing in more than one database. After reviewing the title and abstract, 235 studies were excluded because they were not related to the topic of the present study. A more detailed review found that 72 studies were suitable. Of these, 52 studies were further excluded due to off-target disease. Some studies were excluded because of poor relevance with ESRD or donors. Eleven studies fulfilled selection criteria for methodological quality analysis. But, six studies were excluded due to insufficient data through manual search. Finally, five studies fulfilled our selection criteria for qualitative evaluation. Pairwise meta-analyses were included in the quantitative meta-analysis (Fig. 1). We conducted a systematic review of these five studies to evaluate experimental differences and topic descriptions (Tables 1 and 2). In the quantitative meta-analysis, the number of patients was 1137 to 119,769 and the follow-up period was 6.8 to 15.1 years.

**Table 1:** Baseline characteristics of included studies

Study	Journal	Study type	Data type (single/multi center, claimed data)	Data base	Studied year (Donor)	Exclusion for donor	Studied year (control)	Standard of matching	Exclusion for control	How to calculate GFR	Matched control	Matching variables	Conflict of interests
Lam et al. 2012 [15]	Nephrol Dial Transplant	Retrospective cohort	Claimed data	Cohort study, Canada	1992–2009	none—all donor	1991.7.1-index date		Genitourinary disease, Diabetes, Hypertension, Cancer, Cardiovascular disease, Pulmonary disease, Liver disease, Rheumatological conditions, Chronic infections, History of nephrology consultation, Frequent physician visits (4 / 2yrs), Failed to see a physician at least once in the two years before the index date	CKD	1:10 matched	Age Sex Index date residence (rural vs. urban) Income	None
										Epidemiology Collaboration (CKD-EPI) equation			
Muzaale et al. 2014 [13]	JAMA	Retrospective cohort	Claimed data	Cohort study, United states	1994–2011	none—all donor	1988–1994(enroll)	Healthy non-donor population	Third National Health and Nutrition Examination Survey (NHANES III)—Answered "Yes" to any of these questions by self-report survey	ESRD	1:1 matched	Age, Sex, Race/ethnicity, Education background, BMI at the time of enrollment, History of cigarette smoking, and SBP	United Network for Organ Sharing Institutional grant support from the National Institutes of Health
										Epidemiology Collaboration (CKD-EPI) equation			
Kim et al. 2019 [16]	Renal Epidemiology	Retrospective cohort	Single center	Hospital data	2002–2015	Missing estimated GFR or abdominal US, A history of cancer, A history of CKD, Proteinuria at baseline, CKD at baseline, Chronic kidney disease, renal tumor, renal cancer on US ;and/or partial or total nephrectomy due to renal dz including Renal TB, renal stone, nephritis, renal tumor	2002–2015		Same as donor exclusion	Epidemiology Collaboration (CKD-EPI) equation	Simple comparison between the two groups		None
Mjoen et al. 2013 [14]	Kidney International	Retrospective cohort	Claimed data	National data	1963–2007	eGFR < 70, Age > 70, Age < 20, BMI > 30, BMI < 17, BP > 140/90, BP medication	1985–1987(enroll)		eGFR < 70, Age > 70, Age < 20, BMI > 30, BMI < 17, BP > 140/90, BP medication, Diabetes, CVD, Reduced general health	Epidemiology Collaboration (CKD-EPI) equation	Simple comparison between the two groups		None
Haugen et al. 2020 [17]	Transplantation International	Retrospective cohort	Single center	Cohort study	1972–2007	Missing BP baseline/at follow-up, Age > 70 years, Use of BP medication, BMI > 30.0 kg/m2, BP > 140/90, CKD-EPI GFR < 70 ml/min/1.73m2, Fasting glucose > 7 mmol/l Comorbidity	1984–2013 (enroll) HUNT surveys HUNT1 (1984–1986) HUNT2 (1995–1997) HUNT3 (2006–2008)		Same as donor exclusion	Epidemiology Collaboration (CKD-EPI) equation	General population		MSD Europe AstraZeneca Boehringer Ingelheim Novo Nordisk Pharma, Lilly Sanofi-Aventis Roche

*BMI* Body mass index, *BP* Blood pressure, *CKD* Chronic kidney disease, *CVD* Coronary vessel disease, *eGFR* Estimated Glomerular Filtration Rate, *JAMA* The Journal of the American Medical Association

**Table 2:** End stage renal disease risk of included studies

	Journal	N for donor	N for control	Follow up for donor	Follow up for control	Definition of CKD or ESRD	ESRD (or CKD) among donor	ESRD (or CKD) among control	Covariates for HR	Unadjusted HR	Adjusted HR	Cause of ESRD (or CKD) for donor	Cause of ESRD (or CKD) for control
Lam et al. 2012 [15]	Nephrol Dial Transplant	2027	20,270	6.9 (median)	6.5 (median)	ESRD: Acute dialysis in follow-up up period (continuous veno-venous or intermittent)	1	14			0.58 (95%CI, 0.08–4.47)		
Muzaale et al. 2014 [13]	JAMA	96,217	96,217	7.6 [3.9–11.5]	15.0 [13.7–15.0]	ESRD: Earliest of initiation of maintenance dialysis, placement on the waiting list, receipt of a living or deceased donor kidney transplant	99	36		2.75 (95% CI, 1.96–3.85)			
Kim et al. 2019 [16]	Renal Epidemiology	1901	32,621	15.1 [1.5–43.9]	24.9 [0.1–26.0]	CKD: estimated glomerular filtration rate of < 60 ml/min/1.73m2 and/or the presence of proteinuria in two or more consecutive visits	3	2969	**Model I**		13.63 (95%CI, 4.39–42.26)		
									Age, Sex, Center, Year of screening exam				
									**Model II**		8.63 (95%CI, 2.78–26.84)		
									+ Baseline eGFR, Smoking status, Alcohol intake, Regular exercise, Education level, Hx of DM, HTN, Medication use of DM,HTN, Dyslipidemia				
									**Model III**		8.72 (95%CI, 2.80–27.12)		
									+ Renal compensation hypertrophy				
Mjoen et al. 2013 [14]	Kidney International	1901	32,621	15.1 [1.5–43.9]	24.9 [0.1–26.0]	ESRD: initiation of renal replacement therapy (dialysis, transplantation)	9	22	Inclusion year, Age, Sex, sBP, BMI, Smoking	18.99 (95%CI, 8.63–41.76)	11.38 (95%CI, 4.37–29.63)	Glomerulonephritis (3) Systemic lupus erythematosus (1) Wegner's granulomatosis (1) ANCA-positive vasculitis (1) Sarcoidosis(1) DM/Nephrosclerosis (2)	Glomerulonephritis (5) Pyelonephritis (4) PCKD (4) HTN (3) DM (1) Amyloidosis (1) Systemic lupus erythematosus (1) Drug induced nephropathy (1) Medullary cystic disease (1)
Haugen et al. 2020 [17]	Transplantation International	1029	16,084	11.3 (mean)	16.4 (mean)	CKD: eGFR < 60 ml/min	216	236		14.31 (95% CI, 9.71–21.08)			

*BMI* Body mass index, *CKD* Chronic kidney disease, *DM* Diabetes mellitus, *eGFR* Estimated Glomerular Filtration Rate, *ESRD* End stage of renal disease, *HR* Hazard ratio, *HTN* Hypertension, *JAMA* The Journal of the American Medical Association, *LKD* Living kidney donor, *PCKD* Polycystic kidney disease, *sBP* systolic blood pressure

### Methodological quality

**Fig. 2:**
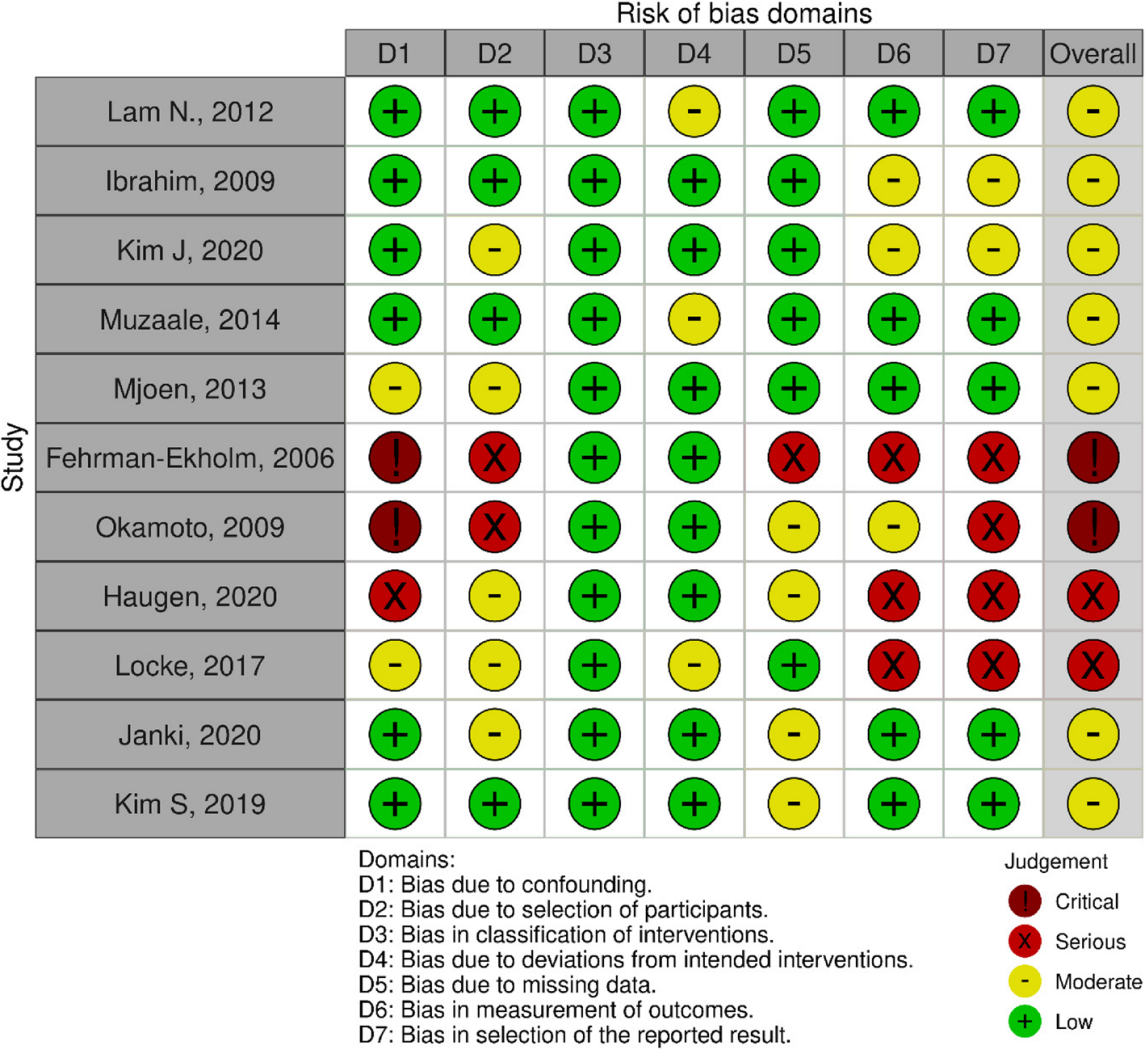
Risk of bias according to ROBINS-I

In each study, seven domains were evaluated using the ROBINS-I tool to determine the risk of bias. In the category of bias caused by confounding domains, the number of ‘Low’ articles was 6, ‘Moderate’ was 2, ‘Serious’ was 1, and ‘Critical’ was 2. In the category of bias caused by selection of participant domain, the number of ‘Low’ articles was 4, ‘Moderate’ was 5, and ‘Serious’ was 2. Classification bias in the interventional domain was ‘low’ because all studies included donor nephrectomy. In the category of bias due to deviations from intended interventional domains, the number of ‘Low’ articles was 8 and ‘Moderate’ was 3. In the category of bias due to missing data, the number of ‘Low’ articles was 6, ‘Moderate’ was 4, and ‘Serious’ was 1. In the category of bias in measurement of outcomes domain, the number of ‘Low’ articles was 5, ‘Moderate’ was 3, and ‘Serious’ was 3. In the category of bias in selection of the reported result, the number of ‘Low’ articles was 5, ‘Moderate’ was 2, and ‘Serious’ was 4. Finally, we determined the overall risk of bias based on results of previous evaluation. As a result for an overall risk of bias, the number of ‘Low’ articles was 0, ‘Moderate’ was 7, ‘Serious’ was 2, and ‘Critical’ was 2 (Fig. 2).

### Outcomes

**Fig. 3:**
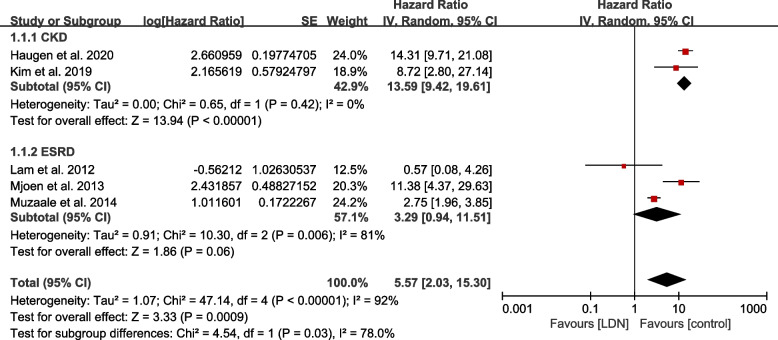
Forest plot of end stage renal disease risk and chronic kidney disease. CI: Confidence interval, eGFR: Estimated glomerular filtration rate, ESRD: End stage renal disease, IV: Inverse variance, LDN: Living donor nephrectomy, SE: Standard error

Detailed results for ESRD risk compared to control groups are described in Fig. 3. In the meta-analysis, the pooled overall ESRD or CKD risk had an HR of 5.57 (95% CI: 2.03—15.30). Cochran’s Q test indicated a high heterogeneity (I^2^ = 92.0%). The pooled ESRD risk had an HR of 3.29 (95% CI: 0.94 – 11.51) and I^2^ was 81.0%. The pooled CKD (eGFR < 60 ml/min/1.73 m^2^) risk had an HR of 13.59 (95% CI: 9.42 – 19.61) and I^2^ was 0%.

### Publication bias

**Fig. 4:**
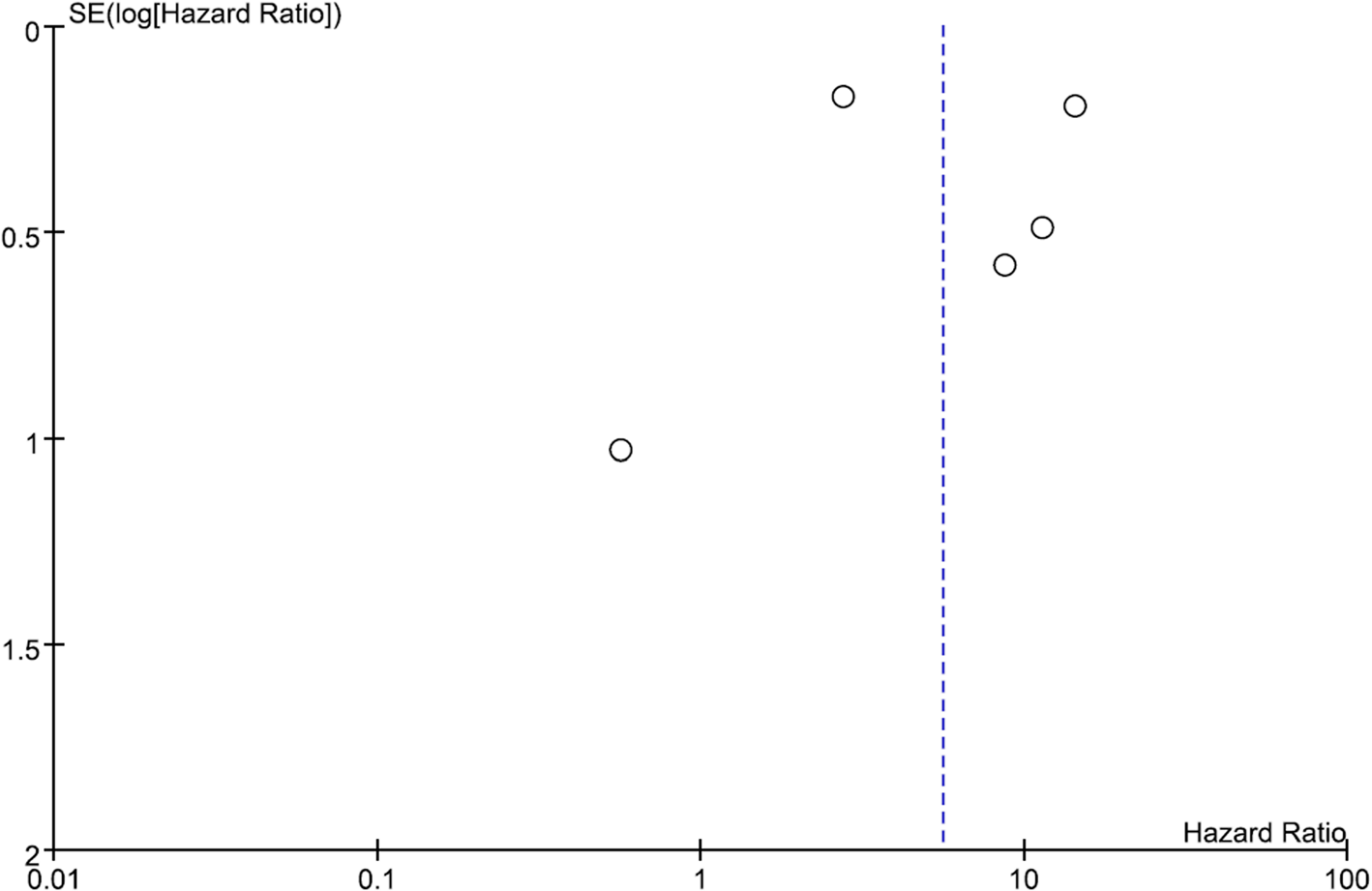
Funnel plot of end stage renal disease risk

Funnel plot of ESRD risk was symmetrical. Results are shown in Fig. 4. *P*-value for Begg and Mazumdar’s correlation test was 0.6242 and Egger’s regression coefficient test was 0.7911. This showed that evidence of publication bias or small-scale research effect did not exist in this meta-analysis.

## Discussion

Our study demonstrated that LDN patients had a higher risk of progression to ESRD compared to the control group (general group and healthy group). We conducted a quality analysis for included studies using ROBINS-I. As a result of overall risk of bias, 7 studies were rated as "moderate", 2 studies were rated as "Serious", and 2 studies were rated as "Critical".

In long-term follow-up, incidence of ESRD in donors is 0.04% to 0.5%, [18, 19] which is lower than that of the general population. Several studies showed similar or much better survival [12, 20] than the general population, because a healthy people with no comorbidities were selected by donors. This contradicts previous reports that kidney donors do not have CKD [9, 21, 22]. One reason for this is that many studies that used the MDRD formula to calculate reported the mean eGFR of the entire cohort rather than the CKD categories. All studies including our analysis calculated eGFR by the CKD-EPI equation. It is suggested that the newly developed CKD-EPI equation for GFR estimation is more accurate than the MDRD equation, especially when GFR is high [23]. It also has fewer biases, better precision, and better accuracy [23].

A reduction in eGFR after LDN is an inevitable result for donors [24]. The risk of renal failure in solitary kidney has been studied extensively over the past 30 years [16]. Brenner et al. have suggested that renal ablation can result in progressive glomerular damage to the remaining kidney associated with glomerular hypertrophy, hyperfiltration, and systemic hypertension [25]. These are related to increases of proteinuria and blood pressure [9, 11]. However, there are also more recent articles around adaptation of the remaining kidney after donation away from glomerular hypertension. Lenihan et al. showed that adaptive hyperfiltration after LDN can be induced by hyperperfusion and hypertrophy of the remaining glomeruli and argued against the progression of significant glomerular hypertension following LDN [26]. Since these factors can increase the risk of cardiovascular and all-cause mortality in the general population [27–29] and kidney donors after nephrectomy [14], it is important to evaluate renal function of donors before and after LDN.

Some studies have evaluated renal function after donor nephrectomy [24, 30] considering the following factors: age, gender, preoperative serum uric acid level, and pre-donation eGFR [24]. With increasing age, renal cortical volume decreases with decreasing GFR, whereas medullary volume increases, balancing the effect of reduced cortical volume on entire kidney volume to some extent [31]. Microscopically, aging in kidney is characterized by nephrosclerosis, for example, increasing focal and global (not segmental) glomerulosclerosis (FGGS), interstitial fibrosis/tubule atrophy, and arteriolosclerosis [32]. The mechanism by gender difference in association between single kidney and risk of CKD has not yet fully identified, but several studies showed the risk of CKD by gender differences [16]. Some studies have demonstrated that estrogen has an antioxidative effect and might protect the kidney through the renal nitric oxide system by weakening oxidative stress or by its effect on components of the renin–angiotensin system [33, 34]. The renal functional reserve (RFR) represents the difference between baseline eGFR and peak eGFR after experiencing difficult situations such as acute kidney injury, pregnancy, and post-nephrectomy state [35]. GFR can maintain normal ranges until 50% of nephron is lost or in patients with a single kidney. So, the RFR test can be a sensitive and early method for evaluation of decreased renal function [35]. Kim et al. [24] also reported that eGFR before donation, quartile range of eGFR percent change after 1 month of donation, and age are important factors associated with long-term renal function results after LDN. In addition, renal functional reserve, indicated through changes in eGFR percentage after 1 month of donation had a greater effect on renal outcomes in patients with lower eGFR before donation than in those with higher eGFR before donation [24]. Therefore, patients with low eGFR should be strictly observed by evaluating their renal functional reserve before donation [36] and with regular checkup after donation. A systematic follow-up program and active examination are needed after transplantation, and closer follow-up is needed for risk groups.

Our study was the first meta-analysis that evaluated ESRD risk in living donor nephrectomy. We conducted a study of individual controls containing healthy groups that met living kidney donor criteria and a comparative study examining various variables. Also, the methodological quality of included studies was evaluated using the ROBINS-I tool. ROBINS-I is a professional tool for assessing risk of bias in non-randomized studies of interventions. There are seven domains including signal questions that provide information related to the determination of each domain which includes confounding, selection of participants, classification of intervention, deviation from intended intervention, missing data, measurement of outcomes, and selection of the reported result. ROBINS-I requires considerable review content and methodology [37].

Despite several advantages, our study has some limitations. Firstly, the design of meta-analysis was based on retrospective studies and the level of evidence was bound to be low because it included only retrospective studies. Secondly, analysis based on variable factors such as age, gender, preoperative serum uric acid level, and pre-donation eGFR was not performed due to insufficient information available. Effects of variable factors on ESRD were not investigated either. Third, outcome of included 2 studies were eGFR less than 60. High levels of within-group heterogeneity and uneven covariate distribution among groups were present. Because the number of included studies was small, studies on the prevalence of CKD were included to confirm the tendency towards ESRD. Fourth, studies had medium term because most of the observational studies have a short observation period.

## Conclusion

Results of this study are consistent with results of recent studies showing an increase in ESRD morbidity in LDN patients. Thus, this study supports the hypothesis from recent studies that ESRD morbidity is increased in LDN patients. However, in our meta-analysis, variables in included studies were not uniform and the number of included studies was small. In order to have a definitive conclusion, meta-analyses of well-planned and detailed studies need to be conducted in the future.

## Supplementary Information

**Additional file 1.** PRISMA 2020 Checklist.

## Data Availability

All data generated or analysed during this study are included in this published article.
